# Two Dimensions of Social Exclusion: Economic Deprivation and Dynamics of Loneliness During Later Life in Europe

**DOI:** 10.1093/geroni/igaa057.2065

**Published:** 2020-12-16

**Authors:** Michal Myck, Charles Waldegrave, Lena Dahlberg

**Affiliations:** 1 Centre for Economic Analysis, CenEA, Szczecin, Zachodniopomorskie, Poland; 2 Family Centre Social Policy Research Unit, Lower Hutt, Wellington, New Zealand; 3 Aging Research Center, Karolinska Institutet, Solna, Stockholms Lan, Sweden

## Abstract

We contribute to the discussion on social exclusion by examining the relationship between material conditions and loneliness in a sample of individuals aged 50+ in the Survey of Health Ageing and Retirement in Europe (SHARE). In its 5th wave, the survey was extended to include specific items related to economic and social deprivation. We use this extended information on material conditions and examine how it correlates with the level and dynamics of a composite loneliness measure at the time of wave 5 and between wave 5 and 6 of the survey (undertaken in 2013 and 2015, respectively). In order to isolate the effect of material deprivation on loneliness, regression analyses include an extensive set of control variables. The analyses show a strong and significant relationship between material deprivation and both the level of loneliness and deterioration in the loneliness status.

